# Convergence effect of the Belt and Road Initiative on income disparity: evidence from China

**DOI:** 10.1057/s41599-022-01315-0

**Published:** 2022-09-12

**Authors:** Bo Qin, Dongmei Zeng, Angang Gao

**Affiliations:** 1 School of Economics, Guangxi University, Nanning, China; 2 School of Economics and Management, Guangxi University of Science and Technology, Liuzhou, China

**Keywords:** Economics, Economics

## Abstract

The international economic effects of the Belt and Road Initiative (BRI) have received much attention, but few studies have focused on the impact of the BRI on domestic regional income disparities. Here, a theoretical framework is proposed based on the logic of public policy analysis in economic geography aiming at studying the impact of the BRI on the convergence of inter-city income disparities in China. Specifically, taking the BRI as a quasi-natural experiment, the impact of the BRI on the convergence of inter-city income disparities in 26 provinces of China is studied empirically using the difference-in-differences method. We find that the BRI has indeed contributed to the convergence of regional income disparities, and this convergence effect is continuously dynamic in its nature. The effects of trade opening and industrial structure transformation are the paths through which the BRI contributes to the convergence of income disparities. Furthermore, we find that there is significant heterogeneity in the effects of the BRI on the convergence of income disparities among cities in different provinces in China. The convergence effect of the BRI on the income disparities among cities in East China is small and insignificant, whereas it can significantly reduce the income disparities among cities in Central and West China. The research in this article has important application value for exploration of the regional income distribution effect of the BRI.

## Introduction

Curbing the continuous expansion of regional income disparity is one of the core issues in regional economy research. China has experienced rapid economic growth over the past 40 years. However, it is undeniable that the income disparity among Chinese residents shows a continuous trend of widening, with the Gini coefficient increasing from 0.288 at the beginning of the reform and opening up to 0.467 in 2017, consistently exceeding the cautionary level of 0.4 (Ravi and Zhang, 1999; Xu and Li, 2009; Li and Zhu, 2018).

The regional income disparity is a key manifestation of the overall income disparity in China (Zhao and Li, 2007; Guan et al., 2019). The continuous expansion of regional income disparity will adversely affect multiple areas of the social economy. First, excessive regional income disparity will easily lead to increased social discontent, which will in turn affect social order, increase the incidence of potential crimes, and be detrimental to maintaining social stability (Elder and Holland, 2000; Hu and Hu, 2007). Second, regional income disparity will further increase the difficulty of overleap the “middle-income trap” and significantly hinder economic growth. On the one hand, income disparity inhibits investment effects and further reduces the level of domestic demand (Persson and Tabellini, 1994; Song et al., 2018; Luo et al., 2019); on the other hand, excessive income disparity can vastly inhibit social entrepreneurship and reduce the dynamism of economic growth (Xu, 2019). Finally, the deterioration of income disparity will seriously hinder the improvement of regional innovation. The larger the income disparity is, the starker the differentiation of income class will be, which will result in the differentiation of consumption levels and hinder the upgrading of consumption structure and is not conducive to the improvement of innovation levels in the long run (Foellmi and Zweimuller, 2017; Cheng and Zhang, 2018).

Therefore, how to promote the convergence of income disparity and thus reduce the negative effects of income disparity has extensively attracted the attention of scholars. The “recipes” prescribed in the existing literature around this issue have focused on the following four aspects: First is the factor endowment theory, whose main argument is that enhancing the regional physical capital stock, especially the level of human capital, can help curb the persistent widening of the interregional income disparity (Mankiw et al., 1992; Gao, 2011; Du and Zhang, 2018; Jiao and Bai, 2020). Second is the market integration theory, which holds that market segmentation is the main cause of regional income disparity, emphasizing that improving the level of market integration by reducing local protection and strengthening inter-regional transportation connectivity is an important step in reducing regional income disparity and alleviating the problem of uneven development among regions (Young, 2000; Xue and He, 2016; Su, 2018; Hong and Wang, 2019). Third is the government factor theory. This theory, on the one hand, holds that government support should be strengthened in the public sector, and the government can be relied upon to effectively reduce the regional income disparity by increasing expenditure on education and social security (Sylwester, 2003; Zhang, 2020; Konstantinou et al., 2021); on the other hand, it holds that breaking the government’s administrative monopoly is the fundamental way to curb the worsening income disparity (Chu and Jin, 2013; Chen and Liu, 2013; Qiu, 2014). Fourth is the knowledge spillover effect. This theory holds that strengthening interregional innovation factor sharing and improving interregional collaborative innovation to broaden the spatial scope of knowledge spillover are important drivers of interregional income convergence (Huang and Zhang, 2010; Magrini, 2014; Zhang and Zuo, 2018). However, few studies have focused on the impact of the BRI on regional income disparity.

As an economic integration strategy to promote global economic welfare, the BRI will have a profound impact on the global economy and China’s economic development. Since its introduction, there has been a wealth of literature on the BRI. The relevant studies mainly focus on two aspects. The first aspect is the impact of the BRI on the level of China’s economic opening to the outside world. Specifically, the BRI has promoted the opening up of China’s border ports (Zhang, 2016; Xuan and Guo, 2020; Xue et al., 2021), boosted Chinese enterprises’ outward direct investment (Liu and Wu, 2018; Long, 2021; Zhou et al., 2022), vastly facilitated the transformation and upgrading of China’s import and export trade (Qin, 2017; Zhang and Cui, 2019; Qiu et al., 2021; Jiang and Duan, 2021). The second aspect is the impact of the BRI on the investment, trade, and economic growth of other BRI countries (Wang and Tan, 2019; Xu et al., 2021; Zhang and Wu, 2022). Most of these studies have focused on the international economic effects of the BRI. The BRI will be a major strategic design that will affect China’s future regional economic geography (An, 2015; Chen and Zhu, 2017; Duan and Wu, 2018; Wu et al., 2018). However, few in-depth studies have explored the intrinsic relationship between the BRI and China’s regional economic geography, the research on the impact of the BRI on the regional economic geography with regional income disparity as the core manifestation has been neglected.

In light of this, we attempted to answer the following four specific questions: First, how does the BRI, as a major policy innovation for China to promote global economic governance in the new era, affect regional income disparities? Second, is the BRI conducive to the convergence of inter-city income disparities? Third, if the BRI helps to curb the widening of regional income disparity, then, what is its mechanism? Fourth, since there is significant regional heterogeneity among different provinces in East, Central, and West China, does regional heterogeneity also exist in the impact of the BRI on the convergence of regional income disparities?

The marginal contributions of the article can be as follows: First, the article focused on the impact of the BRI on the income disparity among cities in a country, which makes up for the lack of research on the economic geography effect of the BRI in the existing literature and provides new empirical evidence for the study of the regional income disparity. Second, we incorporate the impact of the BRI on regional income disparities into the framework of public policy analysis in economic geography, which provides empirical support to enhance the intersection between policy issues and the discipline of spatial geography analysis, and broadens the dimensions of public policy analysis in economic geography. Third, we found that the BRI reduces the income disparity among cities obviously, and the convergence effect is regional heterogeneity.

## Brief review of Chinese economy, income, trade opening, and current policies

### Chinese economy and income

**Fig. 1:**
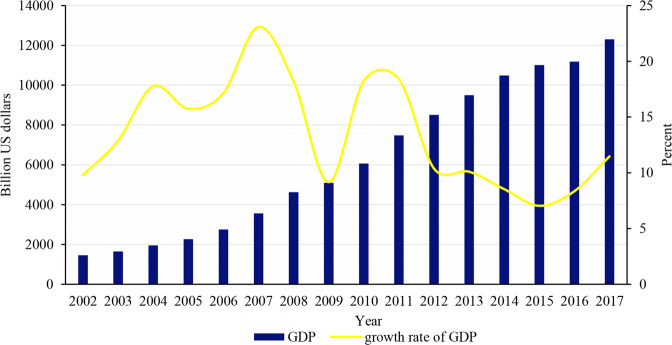
China’s GDP and its growth rate from 2002 to 2017.

**Fig. 2:**
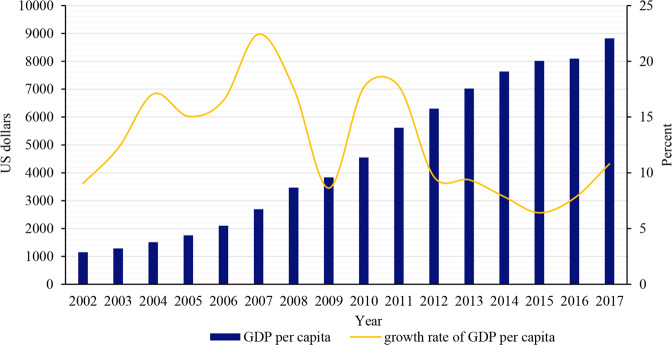
China’s GDP per capita and its growth rate from 2002 to 2017.

China’s economy continued to grow, and its GDP increased from less than 2 trillion US dollars in 2002 to more than 12 trillion US dollars in 2017. Although there was some fluctuation in China’s economic growth rate, especially the economic growth rate in 2009 dropped sharply due to the impact of the global financial crisis in 2008, but China’s economic growth rate is still at the top of the world from 2002 to 2017 in general. With the rapid economic growth, China’s income level has also continued to increase. The GDP per capita has increased from more than 1000 US dollars in 2002 to nearly 9000 US dollars in 2017. Although the GDP per capita growth rate and economic growth showed similar fluctuations trend, it still maintained an average annual growth rate of 13% (Figs. 1 and 2).

**Fig. 3:**
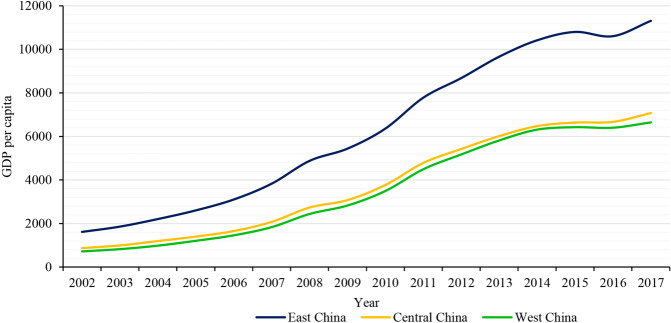
China’s income level in East,Central and West provinces from 2002 to 2017.

The income disparity among provinces in China still exists and has a trend of continuous expansion. In particular, the income disparity between Eastern China and Midwest China continues to widen (Zhou, 2012; Liu et al., 2022). The evolution of income levels among Eastern, Central and Western China can also be seen that the income disparity between Central China and Western China is relatively small, but the income disparity between Eastern China and Midwest China tends to expand over time (Fig. 3).

### Trade opening

**Fig. 4:**
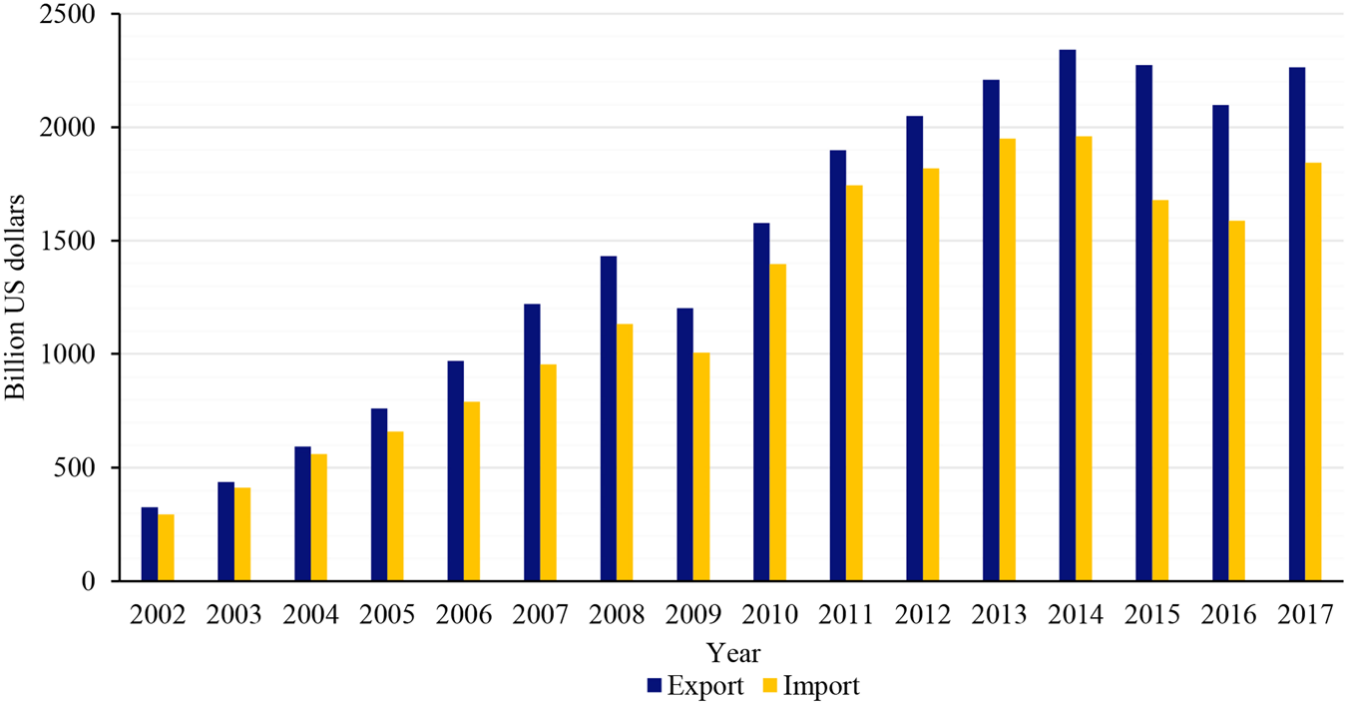
China’s export and import from 2002 to 2017.

**Fig. 5:**
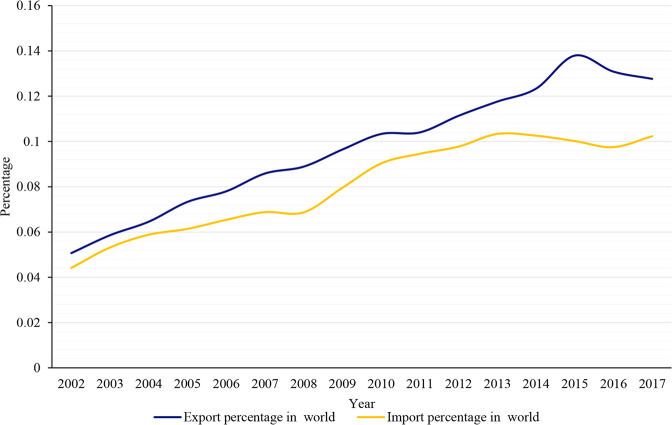
China’s export and import percentage in world from 2002 to 2017.

Since China’s entry into the World Trade Organization in 2001, China’s trade openness has gradually improved. China’s international trade showed a relatively rapid growth overall both in export and import from 2002 to 2017, and exports were more than imports resulting in a steady trade surplus (Fig. 4). In addition, China’s imports and export accounted for more than 10% of the world trade in 2017 and was also showing a rapid upward trend (Fig. 5). China’s trade opening is playing an increasingly important role in global trade growth.

### Current policies

**Fig. 6:**
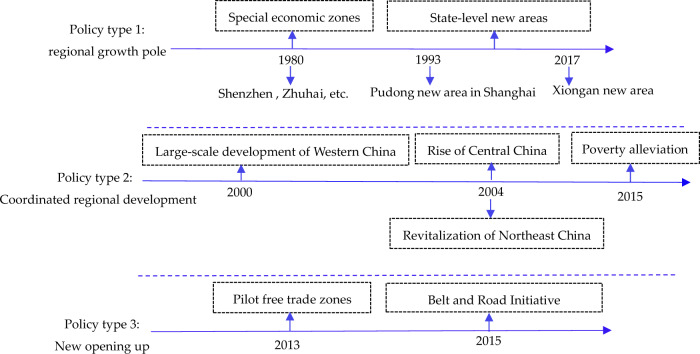
Current policies of China: regional growth pole,coordinated regional development and new opening up.

Since the implementation of the reform and opening-up policy in 1978, China has formulated and implemented many economic policies which can be generally classified into three types according to the research objectives of this paper (Fig. 6). One is the regional growth pole strategy represented by special economic zones and state-level new areas. China implemented the special economic zone system in 1980, and successively established special economic zones in Shenzhen, Zhuhai, Shantou, Xiamen and Hainan Province. In addition, China has also implemented a national-level new area policy to create economic growth poles, such as Shanghai Pudong New Area established in 1993 and Xiongan New Area established in 2017. The second one is a coordinated regional economic development strategy. In order to solve the problem of unbalanced regional development brought about by the regional growth pole strategies, China implemented the strategies of large-scale development of Western China in 2000, rise of Central China in 2004, revitalization of Northeast China in 2004, and the poverty alleviation strategy in 2015. The last one is a new opening policy which mainly includes the pilot free trade zones implemented in 2013 and the BRI formally proposed in 2015.

## Theoretical analysis and research hypothesis

### The BRI and regional income disparity

How does the BRI affect regional income disparity? This is the first theoretical question that this study sought to answer. We argue for the intrinsic relationship between the two based on the policy analysis framework in economic geography.

Throughout the development of different schools of economic geography, public policy research has gradually become an important part of economic geography research. In the 1950 s, based on economic problems, “regionalism” focused on how to propose new public policies to solve real economic problems. By the 1980 s, economic geographers, represented by Harvey (1985), transferred the focus of their research to the economic effects of political and economic systems, while the school of new economic geography, represented by Krugman (1991), emphasized the impact of non-economic factors (such as public policies) on economic outcomes. After the late 1990 s, policies of regional integration and economic globalization pursued by different countries have profoundly affected economic geography patterns at the global spatial scale, prompting the integration of public policy analysis into the theoretical framework of economic geography analysis (Hudson, 2005; Hannum and Wang, 2006; Busso et al., 2013), which, in turn, led to the formation of public policy geography. The geographic effects of social public policies have been the core focus of public policy geography as an important branch of economic geography, emphasizing the geographic connotations of public policies (Martin, 2001; Mi and Xu, 2010). In other words, public policy geography contends that national public policies will significantly influence the differences of economic variables in different geographical spaces and their evolutionary patterns and, on this basis, emphasizes that public policies are an important way to reduce regional differences effectively. The policy assessment of the geographic effects of public policies has gradually become the mainstream in the field of economic geography policy to identify the impact of public policies on geographic effects (Kuus, 2011; Betz and Partridge, 2013), so as to establish an intrinsic link between public policies and economic geography effects.

Regional economic spatial disparity, as one of the most traditional and typical geographical effects, manifests itself in the form of income disparity between regions (Anselin, 2010; Feng et al., 2015). Based on the above analysis, this study argues that the BRI, as a strategically important public policy at the national level, also has economic geographic effects that may affect the regional income disparity in China. The provincial administrative units along the BRI, as the key areas affected by the BRI, will also transmit those effects to prefecture-level cities within these provincial administrative units, bringing new historical opportunities for industrial growth, employment growth, and income growth of residents in prefecture-level cities. On the one hand, the BRI provides a strong external impetus for provincial administrative units to speed up the improvement of transportation infrastructure among cities within the province and enhance the level of interconnection among cities inside and outside the province, thus promoting urban economic growth. On the other hand, the BRI is conducive to the formation of new urban growth poles within the provincial areas, which, in turn, helps to enhance their capacity to facilitate the development of other small and medium-sized cities, promote the reshaping of urban development axes and urban network systems, and create dynamic support for coordinated urban economic development to effectively prevent the further widening of regional income disparities (Zhou, 2017; Su et al., 2018). Based on this, this study argues that the BRI is an important institutional force to curb the widening of the regional income disparity and promote a balanced development of the regional economy.

Based on the above analysis, the first research hypothesis of this study was thus proposed:

Hypothesis1: *The BRI can vastly reduce regional income disparities*.

### Transmission mechanism: trade opening and industrial structural transformation

The BRI, with openness and cooperation as one of its basic principles, is a new model to guide the reallocation of global economic factors and promote China’s future trade and economic openness (Du and Ma, 2015). Since the promulgation of the initiative, the construction of the BRI has significantly contributed to the strategic upgrading of China’s opening up (Gao and Jiang, 2018). The BRI also specifies measures for BRI provinces to increase their openness to the world, which greatly enhances the enthusiasm of provincial and municipal local governments to carry out the BRI pragmatically. On the one hand, the local governments of relevant provinces and cities have issued relevant development strategic plans. On the other hand, they have continuously improved customs clearance facilitation conditions and established early warning mechanisms for local imports and exports, which effectively reduce the environmental uncertainty of local development and the expected risks of both sides of foreign trade and help enhance the level of trade openness of cities and the attractiveness of the foreign investment. In addition, several demonstration projects such as the China–Vietnam Railway, the China–Thailand Railway, and the China–Russia Eastern Gas Pipeline have been steadily promoted, as promoting infrastructure connectivity is also an important goal of the BRI. The improvement of infrastructure has become a powerful driving force for promoting the development of bilateral trade between BRI countries, particularly for the palpable improvement of the level of trade openness of inland cities in Central and West China, as it helps to strengthen the external ties of BRI cities, effectively reduce trade costs, and improve trade efficiency (Hu et al., 2019). In short, the BRI can vastly advance the level of trade openness.

The existing literature is divided regarding the relationship between trade openness and the regional income disparity, with some scholars arguing that trade openness is an important factor contributing to the widening of the regional income disparity (Wood, 1998; Hu, 2017) and others arguing that trade openness helps to narrow the regional disparity (Borjas et al., 1997). This study supports the latter view. First, based on a classic study by Stolper and Samuelson (1941), due to the comparative advantage of labor-intensive industries in developing countries with relatively abundant labor resources, trade opening promotes the expansion of the exports of labor-intensive products and leads to their price increase, which raises the wages of low-skilled labor and thus reduces the income disparity. Second, trade openness can also increase the income of immobile factors by alleviating the concentration of domestic-regional factors in central cities, which, in turn, reduces the regional income disparity (Fujita and Krugman, 1999). Third, trade openness that promotes the inflow of foreign capital will not only advance the economic growth of the inflowing cities but also have positive spillover effects on the income growth of neighboring cities, which will eventually reduce the income disparity among regions in developing countries (Yao and Sun, 2015). Finally, if the barriers of labor mobility between regions are effectively broken, trade opening can effectively reduce regional income disparities, particularly between cities, and lower urban poverty (Zeng and Jiang, 2014). The above analysis shows that trade opening can help reduce the regional income disparity.

Based on the above analysis, the second research hypothesis of this study was proposed.

Hypothesis 2: *The BRI can vastly reduce regional income disparity through the trade opening effect*.

The promulgation of the BRI also provides a new strategic solution to the long-standing problem of industrial restructuring in China. It has vastly contributed to the transformation of China’s industrial structure in at least three ways: First, the BRI provides new opportunities and new access points for the transformation of the domestic regional industrial structure (Zhang, 2018). Second, the BRI promotes the international expansion of the domestic market scale and, in turn, promotes the technological innovation of enterprises via the market scale effect, thereby driving the overall industrial-structure transformation (Ma and Ma, 2017). In addition, the BRI has greatly enhanced outward foreign direct investment(OFDI) in China from BRI countries, bringing the reverse technology spillover effect of OFDI to China, enhancing the scale and strength of China’s international R&D capital, thus promoting the transformation of China’s industrial structure (Yao, 2017). Based on the above analysis, the BRI has greatly advanced the transformation of China’s industrial structure.

Industrial structure transformation is an important factor affecting regional income disparity, as it can change the social demand for different factors of production, which, in turn, affects the income levels of regional owners of different factors of production, thus changing the income distribution (Lin and Liu, 2003). Therefore, promoting regional industrial structure transformation is a key path to narrowing the regional income disparity in China (Fan and Zhang, 2003). This study argues that industrial structure transformation can help narrow the income disparity among regions based on the following three considerations: First, although China has achieved remarkable results in breaking the urban-rural dualistic economic system since the reform and opening-up, the dualistic economic system has not yet completely changed. According to Lewis’ “dual economy” theory, under the dual economy system, a large amount of surplus rural labor will migrate to cities, which, on the one hand, promotes the industrial structural transformation characterized by the rationalization of industrial structure and, on the other hand, raises their income level, which helps to improve the distribution of income and narrow the income disparity. Second, in the context of China’s current aging trend, the industrial structural transformation helps to attract migrants to return to their home villages, which, in turn, encourages different regions, particularly less developed areas, to strengthen human capital investment and improve human capital, thus creating conditions for promoting the coupling between industrial structure transformation and human capital, advancing the economic growth of backward areas, continuously increasing the economic surplus available for distribution, and inhibiting the further expansion of the income disparity among regions (Zhang and Zhang, 2019). Third, a large body of empirical studies suggested that the higher the level of industrial structural transformation was, the smaller the regional income disparity would be. For example, Stark et al. (2009) found that labor mobility in the tertiary sector significantly reduces the income disparity. Wu et al. (2018) found that industrial structural transformation, characterized by advanced industrial structure, has vastly reduced the inter-regional income disparity since the 2008 financial crisis by using Chinese microdata. Based on this, this study argues that industrial structure transformation helps to reduce the regional income disparity.

Based on the above analysis, the third research hypothesis of this study is proposed.

Hypothesis 3: *The BRI can also significantly reduce regional income disparity through the effect of industrial structural transformation*.

**Fig. 7:**
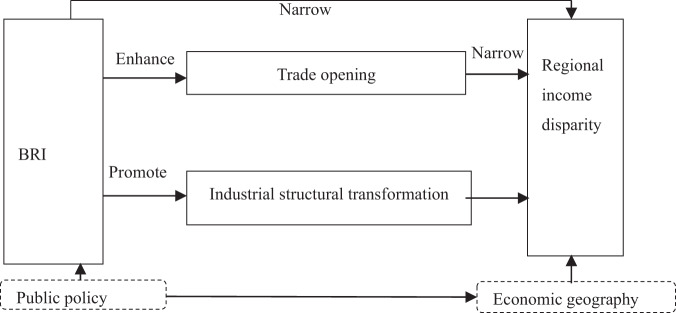
Theoretical framework of the impact of BRI on income disparity: effect and transmission mechanism.

Based on the above analysis, this study constructed a theoretical framework of the effect of the BRI on regional income disparity based on the perspective of public policy analysis in economic geography and theoretically explains the inner mechanism of the influence of the BRI on regional income disparity (Fig. 7), so as to lay the foundation for the empirical analysis in this study.

## Research design

### Sample and data

This study selected panel data of 269 cities in 26 provinces in China from 2002 to 2017 as the sample. As the adjustment of prefecture-level city zoning was relatively frequent in the years before 2002, 2002 was selected as the starting year of the study to ensure the continuity of the study area. The 269 cities in 26 provinces in China were selected for two reasons: First, the BRI has made strategic planning for the functional positioning of the relevant provinces, which significantly affects their provincial economic growth and, in turn, the income disparity among cities in the provincial area. Since this study focused on the income gap between cities within provinces, four municipalities directly under the central government, namely Beijing, Shanghai, Tianjin, and Chongqing, are excluded. Second, since there is only one prefecture-level city in Tibet and Qinghai published in the China Urban Statistical Yearbook, the income gap between cities within provinces could not be calculated. In addition, the data of related variables are missing; thus, these samples were excluded. Third, cities in other provinces and regions with missing data for related variables were excluded. The final sample of 269 cities was obtained, and a quasi-natural experiment of the BRI was conducted to obtain balanced panel data with the number of sample observations standing at 4304.

The data for this study were mainly obtained from China City Statistical Yearbook 2003–2018. China City Statistical Yearbook 2018 only counted the gross domestic product (GDP) at the municipal district level, not the city-wide GDP of each prefecture-level city or the city-wide GDP per capita; therefore, the GDP per capita of each city for 2017 was obtained from the statistical yearbook of the relevant province for 2018.

### Variables

#### Dependent variables

Dependent variable is the regional income disparity (citydis). Most of the existing literature used GDP per capita as an important indicator to reflect regional income level (Liu et al., 2017), and this study also used GDP per capita as an indicator to measure regional income level. There are several indicators that can be used as measures of inequality such as standard deviation, coefficient of variation (CV), Gini coefficient (Gini), and Theil index (Theil). CV and Gini coefficient are used as indicators for measuring income disparity in this study.

CV is selected as an indicator to measure income disparity for the following reasons: Firstly, the standard deviation and Theil index have some limitations. Although the standard deviation can reflect the degree of dispersion of the related variables of each spatial unit within a population, it is an absolute indicator. It lacks comparability when the population averages are different. For example, the average income disparity between Guangdong and Guangxi provinces is relatively large. If the standard deviation is used to measure the internal income disparity in the two provinces, it is possible that the standard deviation is the same. Therefore, it cannot be simply considered that the internal income disparity in the two provinces is the same. Since CV equals standard deviation divided by the mean, this defect can be effectively overcome, so that it can better reflect the income gap among spatial units within a population. Secondly, the functional form of the Theil index determines that the decomposition by revenue source is not possible. As a result, the existing related studies were always carried out independently and not within the same framework of a model. This defect makes the measurement results decomposed by groups inconsistent with the measurement results decomposed by income sources, and further comparative analysis cannot be done. In addition, the Theil index is easily affected by the size of the sample, and the calculation is relatively complicated. Thirdly, CV satisfies the conditions of dimensionlessness, homogeneity, symmetry, and Pigou–Dalton conditions which are necessary for the selection of methods to measure disparity. Fourthly, the CV can also realize the unity of group and source decomposition, and the calculation is simple and easy to understand. Fifthly, existing literature on income disparity used CV to measure the income disparity among regions (Williamson, 1965; Shorrocks, 1982; Akita and Miyata, 2010), especially a large number of scholars used this method to study the income disparity in China (Qin et al., 2011; Yang et al., 2012). Existing studies provide empirical evidence for this study using CV to measure income disparity. Finally, this paper focuses on the income disparity among cities in different provinces in China. The number of cities in each province is definite, and the CV method is very suitable for the objective need of this study.

The specific calculation formula of CV is as follows:

1 $${\mathrm {CV}} = \frac{1}{{\overline {{\mathrm {pgdp}}} }}\sqrt {\mathop {\sum}\limits_{i = 1}^n {\left( {{\mathrm {pgpdp}}_i - \overline {{\mathrm {pgdp}}} } \right)^2/\left( {n - 1} \right)} }$$

where CV denotes the coefficient of variation, the larger the value, the larger the income gap between regions. *n* is the number of cities corresponding to the provincial scale, pgdp is the GDP per capita of city *i*, and $\overline {{\mathrm {pgdp}}}$ is the average GDP per capita of cities within the provincial area.

In addition, Gini coefficient is also dimensionless indicator which was used to measure regional income disparity in related literature (Dagum, 1997; Osberg and Xu, 2000). In order to enhance the robustness of the research conclusions, we also use Gini coefficient to measure the income disparity. The specific calculation formula of it is as follows:

2 $${\mathrm {Gini}} = \frac{1}{{2n^2\mu }}\mathop {\sum}\limits_{j = 1}^n {\mathop {\sum}\limits_{i = 1}^n {\left| {{\mathrm {pgdp}}_j - {\mathrm {pgdp}}_i} \right|} }$$

where Gini denotes the Gini coefficient, the larger the value, the larger the income disparity among regions. *n* is the number of cities corresponding to the provincial scale, *i* and *j* denotes city, pgdp is the GDP per capita of city, and *μ* is the average GDP per capita of cities within the provincial area.

#### Independent variable

The core independent variable is the dummy variable of the BRI, which was promulgated by China in March 2015 as “Vision and Actions on Jointly Building Silk Road Economic Belt and 21st-Century Maritime Silk Road.” The BRI clearly specified the provinces that are to be affected, and that the impact on the income disparity among cities in different provinces may vary, which provides us with the possibility to distinguish the treatment group from the control group. In light of this, the BRI has provided a quasi-natural experiment for this study. With regard to the setup of the experimental and control groups, we followed the practice of Wang and Lu (2019) to set the 14 provinces affected by the BRI as the experimental group and cities in the remaining provinces unaffected by the initiative as the control group. In terms of the independent variable, the dummy variable “treat” was constructed according to whether or not the provinces were affected by the BRI, and the cities in the provinces affected by the BRI were treated as the experimental group and assigned a value of 1, whereas cities in the remaining provinces unaffected by the BRI were treated as the control group and assigned a value of 0. On this basis, the dummy variable “policy*”* was set according to the implementation year of the BRI, and the “policy*”* of 269 cities in 26 provinces in the year of the BRI implementation and beyond was assigned a value of 1, whereas the “policy*”* before the BRI implementation was assigned a value of 0. The interaction term “treat × policy*”* was the core independent variable in this study, and its coefficient sign was used to assess the impact of the BRI on regional income disparity.

#### Mediating variables

According to the research hypothesis, the BRI reduces the regional income disparity through the effect of trade opening and industrial structure transformation. Trade openness (traopen) and industrial structure transformation (transition) were the two mediating variables in this study. The indicators for measuring trade openness were the ratio of total imports and exports to GDP and the ratio of foreign direct investment to GDP, etc. Considering the data for total imports and exports of prefecture-level cities were not easily available, the ratio of foreign direct investment to GDP was used as an indicator to measure the degree of trade openness.

Industrial structure transformation refers to the process of the regional industrial level evolving from a lower level to a higher level. According to the Petty–Clark Theorem, industrial structure transformation is mainly reflected in the increase of the proportion of non-agricultural industries. Around this definition, the industrial structure similarity coefficient, Moore structure index, and the proportion of tertiary industry output value have become the main indicators for measuring industrial structure transformation. With the continuous improvement of urbanization and information technology, the increase in the proportion of the tertiary industry is the key to industrial structure transformation and promoting quality economic growth. In view of this, this study used “the proportion of tertiary industry output value to secondary industry output value” as an indicator to measure industrial structural transformation.

#### Control variables

The control variables mainly included the level of government expenditure on science and technology, the level of urbanization, human capital, the level of marketization, cultural capital, and the degree of informatization. The level of government expenditure on science and technology (govtec) was expressed by the proportion of urban expenditure on science and technology to fiscal expenditure. The level of urbanization (urban) was expressed as the proportion of the population in the urban area to the total population. Human capital (human) was expressed as the number of students in higher education as a percentage of the total population of the city. Market (market) was expressed by the number of private sector employees in urban areas as a proportion of the total number of employees. Cultural capital (culture) was expressed as the number of public books per capita. The degree of informatization (ln net) was expressed as the logarithm of the number of international internet users.

#### Model specification

In studying the effect of the BRI on the convergence of income disparity among cities, the single-difference method can be used to compare the differences in regional income disparity between the two periods before and after the implementation of the BRI to verify the impact of the BRI on regional income disparities. However, the conclusions obtained using the single-difference method may be inaccurate. For cities in different provinces, differences already existed before the BRI was enacted, and the single-difference approach does not take into account such differences, which may lead to an overestimation of the suppressive effect of the BRI on regional income disparities. In the field of policy evaluation research, the difference-in-differences approach has become a common research method (Beck et al., 2010) that can effectively reduce the interference of factors other than policy in the estimation results. In light of this, this study also adopted the difference-in-differences model to identify the convergence effect of the inter-city income disparity in the BRI. Based on the definition of the independent variable, the year 2015, when the BRI was promulgated, was used as the time of the policy shock, and the experimental and control groups were constructed on this basis. The specific econometric model is used:

3 $${\mathrm {citydis}}_{it} = \beta _0 + \beta _1{\mathrm {treat}}_{it} \times {\mathrm {policy}}_{it} + {{{\mathbf{\beta X}}}}_{it} + \gamma _t + \eta _i + \varepsilon _{it}$$

where the subscript *i* denotes the cities in the province, *t* denotes time, and citydis is the dependent variable, which denotes the inter-city income gap in the province in the first year. treat is the city grouping variable, and the value of treat is 1 if the city belongs to the province (autonomous region) significantly affected by the BRI, otherwise the value is 0. policy is a time grouping variable with a value of 0 for policy from 2002 to 2014 and a value of 1 for policy from 2015 to 2017. treat × policy is the interaction term between city grouping and time grouping variables, and *β*_1_ the coefficient of this interaction term is the core parameter in this study, which indicates the impact of the BRI on the regional income disparity. If the exogenous institutional arrangement of the BRI does reduce the income disparity, the coefficient of *β*_1_ should be conspicuously negative. **X** denotes the control variable, *γ*_*t*_ denotes the time fixed effect, *η*_*i*_ denotes the individual fixed effect of each city, and *ε*_*it*_ is the error term.

Considering the lag and time-sensitiveness of policy implementation, the impact of the BRI on the convergence of income disparity among cities may also be nonlinear. In light of this, this study used Model (3) as the basis and set up the following econometric model to capture the income gap convergence effect and its trend for each year after the implementation of the BRI:

4 $${\mathrm {citydis}}_{it} = \delta _0 + \delta _1{\mathrm {treat}}_{it} + \delta _2{\mathrm {policy}}_{it} + \delta _t\mathop {\sum}\limits_{t = 2016}^{t = 2017} {{\mathrm {year}}_t \times {\mathrm {treat}}_{it} + {{{\mathbf{\beta X}}}}_{it} + \gamma _t + \eta _i + \varepsilon _{it}}$$

where year_*t*_ denotes the time dummy variable, which is taken as 2016 and 2017, respectively. *δ*_*t*_ is another core parameter to capture the convergence effect of the income gap between cities and its trend for each year after the implementation of the BRI. The meanings of the other elements in Model (4) are the same as in Model (3).

The descriptive statistics of the main variables are shown in Table 1.

**Table 1:** Variable statistical description.

Variable	Sample size	Mean	Standard deviation	Min	Max
citydis	4304	0.531	0.169	0.038	1.283
treat	4304	0.479	0.499	0.000	1.000
policy	4304	0.187	0.390	0.000	1.000
govtec	4304	0.023	0.047	0.000	0.368
culture	4304	0.490	1.016	0.010	43.249
urban	4304	0.605	1.684	0.033	1.000
ln net	4304	3.181	1.262	−3.744	6.641
human	4304	0.015	0.021	0.000	0.131
market	4304	0.901	0.661	0.024	17.141
traopen	4304	0.020	0.024	0.000	0.419
transition	4304	0.848	0.504	0.094	19.213

## Results

### Univariate analysis

The difference-in-differences model has to satisfy the exogeneity of the policy experimentation on the one hand, and the parallel trend assumption on the other hand. To this end, univariate t-tests were first conducted, and the results are reported in Table 2. Table 2 reports the evolution of the inter-city income disparity before and after the implementation of the BRI. It could be seen that there was a significant difference between the experimental and control groups at the 1% significance level for the income disparity variable before the implementation. After the implementation of the BRI, there was still a significant difference between the experimental and control groups, as evidenced by the fact that the mean value of the income disparity in the experimental group was significantly lower than that in the control group. In addition, the mean values of income disparity variable in the experimental group also changed before and after the promulgation of the BRI; in other words, after the promulgation, the mean values of income disparity variable in the experimental group were lower than before the promulgation. The above analysis shows that the BRI did significantly contribute to the convergence of the inter-city income disparity in the experimental group. However, this convergence effect may also have been influenced by other factors, which need to be analyzed more deeply using a difference-in-differences model.

**Table 2:** Result of univariate *t* test.

Variable		Experiment group (a)	Control group (b)	Difference (a)−(b)	*t*-test (a)−(b)
citydis	Before	0.5139	0.5893	−0.0754	−13.1554^***^
	After	0.4331	0.4745	−0.0414	−4.9860^***^

### Estimation results and analysis of the difference-in-differences model

To further test the average treatment effects and dynamic treatment effects of the BRI on inter-city income disparity, this part of the study applied both the ordinary least squares (ols) and fixed effects (fe) models to perform the difference-in-differences test, and the estimation results are reported in Columns (1)–(4) of Table 3. Columns (1)–(2) represent the average treatment effect of the BRI on the convergence of the inter-city income disparity. Specifically, Columns (1) and (2) show the results of the fixed effects estimation without and with the inclusion of control variables, respectively, and the coefficient of treat × policy which was the core independent variable in this study, was significantly negative at the 1% level. The above results show that the coefficient of the core independent variable treat × policy was significantly negative, regardless of whether the ols model or the fe model was used, which indicates that the BRI has indeed contributed to the convergence of the income disparity among cities.

**Table 3:** Estimation result of the difference in difference.

Variable	Average effect		Dynamic effect	
	(1)	(2)	(3)	(4)
treat × policy	−0.034^***^ (−5.38)	−0.035^***^ (−5.68)		
treat × year_2016_			−0.039^***^ (−3.99)	−0.039^***^ (−4.02)
treat × year_2017_			−0.020^**^ (−2.05)	−0.020^**^ (−2.10)
govtec		−0.351^***^ (−4.40)		−0.339^***^ (−4.25)
market		−0.003 (−1.17)		−0.002 (−0.96)
culture		−0.002 (−1.13)		−0.001 (−1.24)
human		−0.281 (−1.45)		−0.296 (−1.53)
urban		0.001 (0.95)		0.001 (1.01)
ln net		−0.008^**^ (−2.67)		−0.007^**^ (−2.54)
cons	0.432^***^ (26.67)	0.595^***^ (77.42)	0.576^***^ (120.44)	0.594^***^ (77.20)
fixed time	Yes	Yes	Yes	Yes
fixed individuals	Yes	Yes	Yes	Yes
Sample size	4304	4304	4304	4304
Adj-*R*^2^	0.799	0.800	0.311	0.315

*T* statistics or *Z* statistics in parentheses.

***p < 0.01, **p < 0.05.

Columns (3) and (4) of Table 3 report the estimated dynamic treatment effects of the BRI on the convergence of the inter-city income disparity. Columns (3) and (4) show the results of fixed effects estimation without and with the inclusion of control variables, respectively, which indicate that the coefficient of the interaction term between treat and year_2016_, the core independent variable in this study, was significantly negative at the 1% level, and the coefficient of the interaction term between treat and year_2017_ was also significantly negative at the 5% level, regardless of the inclusion of control variables. This indicates that the dynamic treatment effect of the BRI to suppress the widening of regional income disparity showed a continuous trend as the BRI continued to advance, thus verifying Hypothesis 1.

### Endogeneity problem: instrumental variable(IV) approach

The application of the difference-in-differences model requires that the exogeneity condition of policy implementation be satisfied. However, judging from the background of the BRI, the provinces included in the BRI did not satisfy the assumption of randomness, that is, they did not have the condition of exogeneity. Cities in the provinces significantly affected by the BRI did not satisfy the randomness assumption, either. Thus, the sample chosen for this study suffered from policy endogeneity, which in turn affected the robustness of the estimation results. For this reason, further tests are needed to address the endogeneity issue.

The policy grouping variable (treat) and the interaction term (treat × policy) were the endogenous variables in this study. Following the common practice in the literature (Waldman et al., 2008; Acemoglu et al., 2012), this study also adopted the IV approach to address the endogeneity issue. The core of using the IV approach lies in the selection of an appropriate IV. The selection of IV must satisfy two conditions at once: first, the correlation assumption, that is, the selected IV are correlated with endogenous variables; second, the exogeneity assumption, that is, the selected IV are not correlated with the disturbance terms. In light of this, and following Wang and Lu (2019), the provinces along the ancient Silk Road were selected as the IV of the endogenous variables treat and treat × policy mainly for the following reasons: First, the BRI was proposed based on the historical facts of the ancient Chinese Silk Road, and the provinces included in the BRI are basically the same as the provinces along the ancient Chinese Silk Road, that is, there was a strong correlation between the provinces included in the BRI and the provinces along the ancient Chinese Silk Road, thus satisfying the hypothesis that the selected IV are related to the endogenous variables. Second, the ancient Chinese Silk Road is about 2000 years old and has a weak relationship with modern urban economic growth. Therefore, “provinces along the ancient Chinese Silk Road → provinces included in the BRI → inter-city income disparity” becomes the only path of the ancient Chinese Silk Road to affect the inter-city income disparity, which is consistent with the exogeneity hypothesis. Based on this, the provinces along the ancient Chinese can be used as an effective instrumental variable to deal with the endogeneity issue in this study.

With regard to the setup of the IV, the IV of the provinces along the ancient Chinese Silk Road was assigned to 1, otherwise, it was assigned to 0. The two-stage least squares (2SLS) method was used for empirical testing. The endogenous variable was the interaction term treat × policy actually. Thus, when using the provinces along the ancient Chinese Silk Road as the IV the instrumental variable corresponding to the interaction term treat × policy was IV × policy, and the first-stage regression model of the instrumental variable method used was as follows:

5 $${ {treat}}_{it} \times { {policy}}_{it} =\uptheta \left( {{ {IV}}_{it}\times{ {policy}}_{it}} \right) + \beta\,{\boldsymbol{x}}_{it}+\gamma _t + \eta _i + \varepsilon _{it}$$

The regression results of the IV method are reported in Table 4, where Column (1) shows the first-stage regression result and Columns (2) and (3) show the second-stage regression result. From the first stage regression results, it is clear that the coefficient of IV × policy was significant at the 1% level, indicating that the endogenous variables were highly correlated with the IV. For the identification of weak IV, in accordance with Staiger and Stock (1997), the judgment was determined by whether the *F*-statistic was greater than 10. As shown in Table 4, the value of the *F*-statistic was greater than 10; therefore, the issue of weak instrument variables did not exist.

**Table 4:** Effect of the belt and road initiative on income disparity: result of IV regression.

Variables	First stage regression	Second stage regression	
	(1) fe	(2) fe	(3) fe
IV × policy	0.581^***^ (23.17)		
treat × policy		−0.143^***^ (−7.67)	−0.142^***^ (−7.63)
govtec	−0.669^***^ (−3.50)		−0.434^***^ (−5.18)
market	−0.021^***^ (−3.47)		−0.006^**^ (−2.15)
culture	0.015^***^ (4.00)		0.001 (0.08)
human	0.341 (0.73)		−0.177 (−0.88)
urban	−0.002 (−0.87)		0.001 (0.46)
ln net	−0.055^***^ (−7.40)		−0.013^**^* (−3.87)
cons	0.112^*^ (1.95)	0.576^***^ (116.13)	0.604^***^ (74.57)
fixed time	Yes	Yes	Yes
fixed individuals	Yes	Yes	Yes
*F* statistic	20.45		
Sample size	4304	4304	4304
Adj-*R*^2^	0.568	0.258	0.266

*T* statistics or *Z* statistics in parentheses.

***p < 0.01, **p < 0.05, *p < 0.1.

From the regression results of the IV in Columns (2) and (3), it can be seen that regardless of whether control variables were included or not, the regression results using the fixed effects model show that the coefficient of the core independent variables treat × policy, which were the focus of this study were still significantly negative at the 1% level, which fully indicates that after taking into account the endogeneity issue, the BRI still had a suppressive effect on regional income disparity, and the above findings did not change.

Although the regression coefficient becomes larger after addressing the endogeneity problem, it is still significantly negative. The main reasons are as follows: First, this paper deals with the endogeneity problem by selecting IV which can eliminate the influence of omitted variables and disturbance terms on dependent variable partially, and the net effect of independent variable on dependent variable can also be obtained. Therefore, the change of regression coefficient further indicates that the BRI has a strong income disparity convergence effect. Second, the consistency of parameter estimation can be maintained by using IV, which makes the regression coefficient have important reference value. Finally, it’s entirely possible that the IV estimate to be larger than the OLS estimate because IV is estimating the local average treatment effect.

### Robustness test

#### Replacing the dependent variable

The Gini coefficient is also a common indicator to measure income disparity. In this section, the Gini coefficient was used to recalculate the income disparity between cities in the provinces and retest the convergence effects of the BRI on the income disparity. The estimation results are reported in Columns (1) and (2) of Table 5. By comparing the results and magnitudes of the variables in Table 5 with Table 3, it can be seen that the results after replacing the dependent variable show that the direction and significance of the coefficient remain the same as using CV. In other words, the core independent variable in this study was still significantly negative at the 1% level regardless of whether ols or fe was used, indicating that the convergence effects of the BRI still existed after replacing the dependent variable. The findings of the study did not change.

**Table 5:** Robustness test of the model.

Variables	Replacing the dependent variable		Triple difference estimation	
	(1) fe	(2) fe	(3) fe	(4) fe
treat × policy	−0.014^***^ (−4.96)	−0.015^***^ (−5.06)		
tridid			−0.034^***^ (−5.55)	−0.035^***^ (−5.68)
govtec		−0.184^***^ (−4.96)		−0.351^***^ (−4.40)
market		−0.002 (−1.30)		−0.003 (−1.17)
culture		−0.002^**^ (−2.14)		−0.002 (−1.13)
human		−0.307^***^ (−3.42)		−0.280 (−1.45)
urban		0.001 (0.99)		−0.001 (0.95)
ln net		−0.003^**^ (−2.34)		−0.008^***^ (−2.67)
cons	0.307^***^ (138.41)	0.317^***^ (88.78)	0.576^***^ (120.63)	0.595^***^ (77.42)
fixed time	Yes	Yes	Yes	Yes
fixed individuals	Yes	Yes	Yes	Yes
Sample size	4304	4304	4304	4304
Adj-*R*^2^	0.315	0.322	0.313	0.317

*T* statistics or *Z* statistics in parentheses.

***p < 0.01, **p < 0.05, *p < 0.1.

In addition, The CV and Gini both are two typical dimensionless methods to measure income disparity. Although the results have certain differences, they are still comparable. This paper also gives some discussion about it in the section on dependent variables. Compared with the previous regression results, the coefficients after replacing the dependent variable become smaller, but after standardizing the coefficients by using the standard deviations respectively, the results show that the regression coefficient of CV is equivalent to 5.0 standard deviations, and the regression coefficient of Gini is equivalent to 5.2 standard deviations. Therefore, whether the CV or Gini coefficient are used as dependent variables, the values after normalizing the coefficients with the standard deviations are almost the same. Therefore, it indicates that the results and magnitudes of the two dependent variables are basically the same.

#### Triple difference estimation

The existence of non-parallel trends may affect the estimation results of the difference-in-differences model, and the triple difference model is effective in reducing the bias caused by non-parallel trends and has been widely adopted (Garthwaite et al., 2014). The following specific triple difference model was set up:

6 $${\mathrm {citydis}}_{it} = \sigma _0 + \sigma _1{\mathrm {tridid}}_{it} + {{{\mathbf{\beta X}}}}_{it} + \gamma _t + \eta _i + \varepsilon _{it}$$

where tridid denotes the triple difference variable, and the coefficient of tridid is assigned to 0 for cities in provinces unaffected by the BRI before its promulgation (2002–2014) and 1 for cities in provinces affected by the BRI after its promulgation (2015–2017). *σ*_1_, the coefficient of tridid, is the core coefficient of interest in the triple difference model. The estimated results are reported in Columns (3) and (4) of Table 5, where it can be seen that the coefficients of the triple difference variable tridid were almost the same as the coefficients in Table 3 and significantly negative at the 1% level and, indicating that the convergence effects of the BRI on income disparity still existed after the triple difference estimation was used, once again testifying to the robustness of the findings in this study.

#### Propensity score matching

To overcome the problems of non-parallel trends in the difference-in-differences model, propensity score matching was applied to retest the empirical results to obtain a net policy treatment effect. The estimated results are reported in Table 6, which shows that the change in average treatment effect is quite slight and the coefficients are still negative and significant at the 1% significance level, regardless of whether *K*-nearest neighbor matching, radius matching, kernel matching, or Mahalanobis distance matching was applied, which is basically consistent with the previous estimation results and further demonstrates the robustness of the findings in this study.

**Table 6:** Propensity score matching test of the model.

Dependent variable	Match method	Average treatment effect	*t*
citydis	*K*-nearest neighbor	−0.035	−2.80
	radius	−0.030	−3.35
	kernel	−0.035	−3.95
	Mahalanobis	−0.044	−4.75

### Mechanism test

The above empirical results suggest that the BRI has a significant convergence effect on the inter-city income disparity. Based on Hypotheses 2 and 3, this part of the study tested the mechanism via which the BRI impacts the convergence of the income disparity through the effect of trade opening and industrial structure transformation using a specific econometric model. The following econometric model was set up:

7 $$\begin{array}{ll}{\mathrm {citydis}}_{it} = \lambda _1{\mathrm {treat}}_{it} + \lambda _2{\mathrm {policy}}_{it} + \lambda _3{\mathrm {traopen}}_{it}\left( {{\mathrm {transition}}_{it}} \right)\\ \qquad\qquad+\, \lambda _4{\mathrm {treat}}_{it} \times {\mathrm {policy}}_{it} \times {\mathrm {traopen}}_{it}\left( {{\mathrm {transition}}_{it}} \right) \\\qquad\qquad+ {\mathbf{\upbeta}} {\mathbf{x}}_{it} + \lambda _1 + \gamma _t + \eta _i + \varepsilon _{it}\end{array}$$

where traopen and transition denote trade openness level and industrial structural transformation respectively and the other items have the same meaning as in Model (3). The coefficient *λ*_4_ was the third core parameter in this study, which was used to identify whether the BRI converged the income gap through the effect of trade openness and industrial structural transformation.

The regression results regarding the mechanism test are reported in Table 7. Columns (1) and (2) of Table 7 show the regression results of the convergence effect of the BRI on inter-city income disparity through the trade opening effect. As can be seen, treat × policy × traopen, the coefficient of the core independent variable in this section, was significantly negative at the 5% level, regardless of whether the control variables were included, indicating that the BRI did curb the widening of the income disparity by promoting trade openness. Columns (3) and (4) show that the BRI affected the convergence of the inter-city income disparity through the effect of industrial restructuring. It can also be seen that the coefficient of treat × policy × transition, another core independent variable in this section, was significantly negative at the 1% level, regardless of whether control variables were included, which fully indicates that industrial structural transition is also an important path through which the BRI affects income disparity convergence. The above analysis shows that Hypotheses 2 and 3 of this study were verified.

**Table 7:** Result of mechanism test.

Variables	Effect of trade open		Effect of industrial restructuring	
	(1) fe	(2) fe	(3) fe	(4) fe
treat				
policy	−0.109^***^ (−15.96)	−0.088^***^ (−8.33)	−0.100^***^ (−7.11)	−0.078^***^ (−7.24)
traopen	0.718^***^ (9.68)	0.722^***^ (9.44)		
transition			−0.003 (−0.82)	−0.002 (−0.71)
treat × policy × traopen	−0.455^**^ (−2.00)	−0.470^**^ (−2.02)		
treat × policy × transition			−0.032^***^ (−7.11)	−0.032^***^ (−7.16)
govtec		−0.291^***^ (3.67)		−0.354^***^ (−4.45)
market		0.003^**^ (−1.19)		−0.003 (−1.23)
culture		−0.001 (−0.82)		−0.002 (−1.17)
human		0.057 (0.29)		−0.152 (−0.78)
urban		0.001 (0.18)		0.001 (0.96)
ln net		−0.009^***^ (−2.78)		−0.008^***^ (−2.79)
cons	0.557^***^ (108.88)	0.574^***^ (72.90)	0.578^***^ (101.06)	0.597^***^ (71.99)
fixed time	Yes	Yes	Yes	Yes
fixed individuals	Yes	Yes	Yes	Yes
Sample size	4304	4304	4304	4304
Adj-*R*^2^	0.323	0.326	0.317	0.321

### Further study: regional heterogeneity

Regional heterogeneity is a typical feature of China’s economic growth, with significant differences within and among provinces and regions in the three major zones of East, Central, and West China. Even the same policy at the national level has different effects on economic growth in different regions, and the exploration of regional heterogeneity has been one of the main focuses of scholars’ attention (He and Liang, 2004). To this end, this study used a subsample regression to investigate whether heterogeneity also exists in the impact of the BRI on the convergence of income disparities among cities of the provinces in East, Central, and West China. The regression results are reported in Table 8.

Column (1) in Table 8 represents the regression results for regions in East China, while Columns (2) represents the regression results for regions in Central and West China. It can be seen that with the inclusion of control variables, the coefficients of treat × policy for regions in East China, although negative, were not significant statistically. The coefficients of treat × policy for regions in Central and West China were also negative. However, they were significant at the 1% level. This indicates that there is significant heterogeneity in the impact of the BRI on the convergence of income disparities among cities of the provinces in East, Central, and West China. In other words, the convergence effect of the BRI on the income disparity among cities in provinces of East China is not significant, but it can significantly reduce the income disparity among cities in provinces of Central and West China. The convergence effect of the BRI on the inter-city income disparity in provinces of Central and West China is significantly larger than that in provinces of East China.

The heterogeneity of the convergence effects of the BRI on the income disparity among cities in different provinces can be explained as follows: First, the economic environment of each province is different due to the different geographical locations, and the economic policies are also different. In particular, the Central and Western provinces pay more attention to the balanced economic development within the province. Many Central and Western provinces have formulated their in-house growth strategies for regional economic coordination such as building multiple provincial sub-central cities and their own economic zones to provide internal policy support for the convergence of income disparity. For example, Sichuan Province has chosen Mianyang, Yibin, Luzhou, Nanchong, and Dazhou as provincial sub-central cities, and it also has established four economic zones including Chengdu Plain Economic Zone, Northeast Sichuan Economic Zone, Northwest Sichuan Ecological Economic Zone, and South Sichuan Economic Zone.

Second, national strategies such as Rise of Central China and the Large-scale development of Western China can only benefit Central and Western provinces, but not the Eastern provinces. Therefore, these spatial strategies embedded in the Central and Western provinces are gradually internalized as unique advantages for the convergence of income disparity.

Third, from the spatial layout of the provinces significantly affected by the BRI, most of the provinces are located in Central and West China. This allows more cities in provinces of Central and West China to share the economic growth effect brought by the BRI, thus injecting greater vitality into narrowing the income disparity.

Finally, this may be related to the diminishing “marginal policy effect.” It is worth noting that more national strategies for opening up and industrial development are embedded in Eastern provinces than in Central and West provinces. Thus, when relevant Eastern provinces are affected by the BRI, it is equivalent to imposing a “marginal policy” on them. According to the law of diminishing marginal effects in economic theory, the convergence effects of the BRI on the income gap among cities in provinces of East China are relatively weak. However, the “marginal policy” of BRI can have a greater “economic growth” effect in Central and West China. The convergence effects of regional income disparity among cities in provinces of Central and West China are thus stronger.

**Table 8:** Regression result of regional heterogeneity.

Variables	East China (1)	Central and West China (2)
treat × policy	−0.001 (−0.09)	−0.048^***^ (−3.25)
govtec	−0.478^***^ (−3.14)	−0.142 (−1.30)
market	−0.016^**^ (−2.35)	0.005 (1.05)
culture	0.001 (0.07)	−0.001 (−0.19)
human	0.196 (0.46)	−0.582 (−1.04)
urban	−0.005^*^ (−1.89)	0.002^*^ (1.71)
ln net	−0.001 (−0.18)	−0.021^***^ (−2.96)
cons	0.642^***^ (31.92)	0.576^***^ (42.19)
fixed time	Yes	Yes
fixed individuals	Yes	Yes
Sample size	1504	2800
Adj*-R*^2^	0.522	0.265

## Discussion and conclusion

In this study, panel data of 269 cities in 26 provinces in China from 2002 to 2017 were selected as the research sample, and the formal implementation of the BRI was taken as a quasi-natural experiment. Experimental and control groups were constructed based on whether the provinces were significantly affected by the BRI, with the focus on the income disparity among cities in the provinces. The difference-in-differences approach was adopted to investigate the effect of the BRI on the income disparity among cities. The results showed the following: (1) The BRI has indeed promoted the convergence of regional income disparity, and it has a dynamic effect on the convergence of regional income disparity with the continuous promotion of the BRI. After addressing the endogeneity problem and conducting robustness tests, the income disparity convergence effect of the BRI still exists, and the findings of the study remain unchanged. (2) The BRI can greatly contribute to the income disparity convergence among cities through two effects of trade opening and industrial structure transformation. (3) Further study found that significant heterogeneity exists in the impact of the BRI on the convergence of income disparity among cities in different provinces in China. The regression results for the Eastern and Central-Western provinces showed that the BRI has had a nonsignificant convergence effect on the income disparity among cities in the Eastern provinces, but it can significantly reduce the income disparity among cities in the Central-Western provinces. The convergence effect of the BRI on the inter-city income disparity in the Central-Western provinces is palpably larger than that in the Eastern provinces.

The significance of the study is as follows. First, regional income disparity is a common economic phenomenon among different countries or regions in the world. If the income disparity continues to widen, it will not be conducive to social stability and sustainable economic growth, nor will it be conducive to the sharing of the fruits of economic development by the people. Therefore, how to promote the convergence of regional income disparity is a major practical problem that needs to be solved. Second, the convergence of regional income disparity is a basic problem that economic growth and regional economics have always been concerned about. Following this thread, this paper further explores the factors affecting regional income disparity, which is a further application of regional economic theory. Third, the proposal of the BRI has aroused great research interest, and much literature has focused on this topic. Therefore, it is of great significance to study the theme of the BRI. Fourth, the existing literature on the BRI paid attention to the investment effect and trade opening effect of the BRI basically, the economic and geographical effect of the BRI is ignored. It will help to make up for the deficiencies of the existing literature, enrich the literature related to the BRI, and provide new empirical evidence for the study of regional income disparity. Fifth, the research is an exploration of the intersection between the disciplines of public policy analysis and spatial economic geography. In order to lay a theoretical foundation for this study, this study incorporates the impact of the BRI on regional income disparity into the public policy analysis framework of economic geography, providing empirical research support for enhancing the intersection between policy issues and spatial geography analysis. Finally, some policy suggestions which are of decision-making reference value not only for China but also for the formulation of regional economic coordinated development strategies of other countries in BRI are given.

The BRI is a public policy of globalization. Some previous studies suggested that globalized public policy can help to reduce the income disparity. The simulation results showed that globalized public policies could promote the convergence of income disparity in the long run (Boucekkine et al., 2013), and many empirical studies have also found that public policy changes can explain income disparity to a large extent (Cai, 2007; Celeste and Nadanovsky, 2010). However, in the research on the globalized public policy of the BRI, some studies believed that the BRI led to a greater concentration of economic activities in some regions, especially in the BRI economies, so benefits from the BRI will not be shared equally, resulting in a widening regional income disparity (Enderwick, 2018; Bird et al., 2020), which is inconsistent with the findings of this paper and may weaken the research conclusions of this paper to a certain extent. This is mainly because the samples of these two studies are different from this paper, which can lead to different conclusions. However, some findings of existing studies are basically consistent with the findings of this paper. Ma (2022) found that the BRI was more conducive to improving the economic performance of lower-income regions, thereby promoting the convergence of the income disparity among regions. The research of Luo et al. (2020) also found BRI can help narrow the overall income disparity within a country by raising the income level of ordinary workers in the BRI countries. While both studies focused on income disparities among the entire population of the country (which is not the focus of this paper), the general conclusions drawn provide important support for enhancing the arguments of this paper.

Although existing studies on the relationship between trade opening and income disparity found that trade opening reduces labor income share, thereby widening income disparity (Jayadev, 2007; Asteriou et al., 2014), these empirical analyses mainly focused on trade opening among developed economies, while this paper focuses on a developing country. Contrary to the conclusions of these studies, many studies suggested that when countries engage in trade opening, the production factors that are relatively abundant gain, especially in developing countries can gain a larger share of labor income through trade opening, which in turn leads to a decrease in income inequality (Jaumotte et al., 2013; Roser and Cuaresma, 2016; Khan et al., 2020; Dorn et al., 2022). These previous studies further provide empirical support for the rationality of the research hypothesis in this paper. In addition, we also discuss in detail that trade opening can promote the reduction of income disparity through the factor diffusion effect and a spatial spillover effect in the part of theoretical analysis and research hypothesis.

Our research has some limitations. Although the sample employed in this paper serves as a great candidate for the analysis of the convergence effect of BRI on income disparity, we recognize the necessity to expand the sample to other countries for exploring the convergence effect of BRI on income disparity among countries. In addition, COVID-19 may have an impact on the policy direction of the BRI. In this context, how the BRI affects regional income disparity will also be an important research topic in the future.

### Policy recommendations

In view of the restraining effect of the BRI on regional income disparity, the top-level design of the integration of the BRI and the coordinated development strategy of the regional economy should be introduced in a timely manner to add new institutional impetus to narrow regional income disparity. Specifically, it is necessary to actively improve the extensive participation mechanism of the BRI, and constantly expand the response space of the BRI. The connection mechanism between the unaffected provinces and the key affected provinces should be further strengthened and the BRI cooperation network of Chinese provinces should be also actively built to share the income growth dividends from the BRI.

The upgrading of international trade can be promoted to provide the driving force for opening up to curb the widening of regional income disparity by taking advantage of the opening-up opportunities brought by the BRI. Specifically, on one hand, the provincial government can promote the balanced development of trade openness among cities by formulating a coordinated development strategy for the level of trade openness. The trade opening policy should be appropriately inclined towards cities with a lower level of openness, and the spatial spillover effect of trade opening in large cities on small and medium-sized cities should also be exerted. On the other hand, some new measures for trade facilitation should be adapted to strengthen trade cooperation with BRI countries. It is necessary to continuously optimize the urban business environment, and strengthen connectivity with BRI countries in the fields of transportation and culture. In addition, the mechanism for dealing with trade disputes should be improved to reduce trade costs.

Regional industrial structure transformation should be appropriately integrated with the BRI. The BRI provinces can identify their integration point with the international production capacity cooperation mechanism according to their own industrial structure. Scientific and technological cooperation among BRI countries should also be strengthened to promote the interaction of advanced technology and management experience, which can lay the industry foundation for narrowing regional income disparity through the transformation of the industrial structure.

In view of the significant heterogeneity in the impact of the BRI on the income disparity, more attention should be paid to the impact of the BRI on income disparity in the Eastern provinces of China. Make more cities in Eastern provinces share the income growth effect brought about by the BRI for the convergence of income disparity.

## Data Availability

The dataset analyzed during the current study is available in the Dataverse repository: 10.7910/DVN/TABDBD. These datasets were derived from the statistics database of the China National Knowledge Infrastructure (CNKI, https://data.cnki.net/yearbook/Navi). The author’s institution has got permission for reusing CNKI through payment.
